# Cross-tissue correlations of genome-wide DNA methylation in Japanese live human brain and blood, saliva, and buccal epithelial tissues

**DOI:** 10.1038/s41398-023-02370-0

**Published:** 2023-02-27

**Authors:** Shota Nishitani, Makoto Isozaki, Akiko Yao, Yoshifumi Higashino, Takahiro Yamauchi, Masamune Kidoguchi, Satoshi Kawajiri, Kenzo Tsunetoshi, Hiroyuki Neish, Hirochika Imoto, Hidetaka Arishima, Toshiaki Kodera, Takashi X. Fujisawa, Sadahiro Nomura, Kenichiro Kikuta, Gen Shinozaki, Akemi Tomoda

**Affiliations:** 1 Research Center for Child Mental Development, University of Fukui, Fukui, Japan; 2 Division of Developmental Higher Brain Functions, United Graduate School of Child Development, Osaka University, Kanazawa University, Hamamatsu University School of Medicine, Chiba University, and University of Fukui, Osaka, Japan; 3 Life Science Innovation Center, School of Medical Sciences, University of Fukui, Fukui, Japan; 4 Department of Neurosurgery, University of Fukui, Fukui, Japan; 5 Department of Neurosurgery, Sugita Genpaku Memorial Obama Municipal Hospital, Obama, Japan; 6 Department of Neurosurgery, Yamaguchi University School of Medicine, Ube, Japan; 7 Stanford University School of Medicine, Department of Psychiatry and Behavioral Sciences, Palo Alto, CA USA; 8 Department of Child and Adolescent Psychological Medicine, University of Fukui Hospital, Fukui, Japan

**Keywords:** Predictive markers, Epigenetics and behaviour

## Abstract

Neuroepigenetics considers genetic sequences and the interplay with environmental influences to elucidate vulnerability risk for various neurological and psychiatric disorders. However, evaluating DNA methylation of brain tissue is challenging owing to the issue of tissue specificity. Consequently, peripheral surrogate tissues were used, resulting in limited progress compared with other epigenetic studies, such as cancer research. Therefore, we developed databases to establish correlations between the brain and peripheral tissues in the same individuals. Four tissues, resected brain tissue, blood, saliva, and buccal mucosa (buccal), were collected from 19 patients (aged 13–73 years) who underwent neurosurgery. Moreover, their genome-wide DNA methylation was assessed using the Infinium HumanMethylationEPIC BeadChip arrays to determine the cross-tissue correlation of each combination. These correlation analyses were conducted with all methylation sites and with variable CpGs, and with when these were adjusted for cellular proportions. For the averaged data for each CpG across individuals, the saliva–brain correlation (*r* = 0.90) was higher than that for blood–brain (*r* = 0.87) and buccal–brain (*r* = 0.88) comparisons. Among individual CpGs, blood had the highest proportion of CpGs correlated to the brain at nominally significant levels (19.0%), followed by saliva (14.4%) and buccal (9.8%). These results were similar to the previous IMAGE-CpG results; however, cross-database correlations of the correlation coefficients revealed a relatively low (brain vs. blood: *r* = 0.27, saliva: *r* = 0.18, and buccal: *r* = 0.24). To the best of our knowledge, this is the fifth study in the literature initiating the development of databases for correlations between the brain and peripheral tissues in the same individuals. We present the first database developed from an Asian population, specifically Japanese samples (AMAZE-CpG), which would contribute to interpreting individual epigenetic study results from various Asian populations.

## Introduction

The International Psychiatric Genomics Consortium conducted the world’s largest genome-wide association meta-analysis consisting of 37 000 patients with schizophrenia and 113 000 controls and identified 108 genome-wide significant genes [1]. Although discovering genetic factors that contribute to disease pathophysiology was useful, the effect size of each genotype turned out to be diminutive. This made it unlikely that schizophrenia and other psychiatric disorders could be revealed solely based on genetic variations. Hence, the molecular genetic vulnerability of psychiatric disorders based on nature and nurture is being explored which has accelerated the investigation of epigenetics in psychiatric disorders. However, the central issue with epigenetics research on psychiatric disorders is the inability to directly handle the target organ—the brain tissue [2–4]. Because identical genetic sequences can be obtained from peripheral tissues (with exceptions), identifying genetic polymorphisms, such as SNPs, as risk genes for psychiatric disorders using peripheral tissues has not posed a problem. However, because epigenetic signatures such as DNA methylation are tissue-specific [5], brain tissue is ideally required for epigenetics analysis in psychiatric disorders. As brain tissue cannot be obtained from living humans in most circumstances, post-mortem brain research in which brain tissue can be handled has been conducted as an alternative; however, analyses using retrospective data and the sample size are limited [6]. Contrarily, progress has been made in elucidating the cancer epigenome as a result of the direct analysis of resected pathological tissues [7]. This dilemma has slowed the advancement of epigenetics research into psychiatric disorders.

Despite this dilemma, previous studies, which attempted to determine the epigenome of psychiatric disorders, used blood or saliva as surrogate tissues for empirical reasons [3, 8]. These peripheral epigenomes are biologically relevant since they have been used not only as a surrogate of brain tissue, but also for studying epigenetic changes occurring in peripheral tissues themselves, such as in the immune system [9, 10]. However, the interpretation would be tantalizing when using peripheral tissue as a surrogate for the brain because they both have distinct DNA methylation patterns; therefore, an association between peripheral tissue DNA methylation levels and certain phenotypes does not necessarily reflect the effect of DNA methylation status in the brain. Owing to the difficulty in concluding that the epigenetic mechanisms directly responsible for the susceptibility of the psychiatric disorders of interest have been identified, the biomarker aspect must be emphasized in many cases [11]. In such instances, animal studies should typically be conducted for tasks that cannot be performed on humans. However, only a few studies had been conducted on epigenetics in psychiatric disorders in animal models when the research focus is limited to genome-wide study because of the absence of microarray platform for major experimental animals (a microarray chip for mice just recently became available in early 2021). With the new array designed for mice, the epigenome of the mouse brain can be studied directly. However, the human brain epigenome will need to be elucidated to translate the results obtained from mice to humans and determine how they can be extrapolated to humans.

The most promising approach to addressing this issue in human epigenetics research would be to confirm the correlation level with the brain epigenome based on genome-wide methylation correlation databases between peripheral tissues and the brain from the same individuals. Thus, the databases enable users (individual researchers) to determine whether the methylation sites identified in their own studies involving peripheral tissues are reliably correlated with brain methylation level. To date, four such databases have been developed. The first database (https://epigenetics.essex.ac.uk/bloodbrain/) is based on the study conducted by Hannon et al. [12] on 71–75 individuals whose brain tissue was obtained from a brain bank and blood samples were collected prior to their death. This database contains extensive information for each of the four brain regions: the prefrontal cortex (PFC), entorhinal cortex (EC), superior temporal gyrus (STG), and cerebellum (CER). For the first time, this study showed the genome-wide DNA methylation differences and similarities between brain and blood tissues in the same individuals using Illumina 450 K array. This study relied on post-mortem brain samples, which have various limitations, such as limited sample size and a lack of premortem phenotype information [6]. The second database is that of Edgar et al. [13], who published a similar article on the post-mortem brain (BA7, 10, and 20) and blood DNA methylation in 16 individuals using the Illumina 450 K array. They made their database, Blood–Brain Epigenetic Concordance (BECon), publicly available (https://redgar598.shinyapps.io/BECon/). As time progressed and data cleaning methods, which improve the quality of DNA methylation array data became more readily available, their greatest advancement was performing this data cleaning with greater precision than Hannon et al. [12]. Specifically, they eliminated batch effects and accounted for tissue cell proportions [14, 15]. For the third database, Braun et al. [4] examined DNA methylation correlations between living human brains and multiple tissues, such as blood, saliva, and buccal epithelial samples, and created a database, Iowa Methylation Array Graphing for Experimental Comparison of Peripheral tissue & Gray matter (IMAGE-CpG) (http://han-lab.org/methylation/default/imageCpG). Although the brain tissues were limited to surgically resected regions from patients with intractable epilepsy that were not necessarily intact, the use of living human brains distinguishes this study from others that relied on post-mortem brain tissues. Edgar et al. [13] corrected the dataset for the proportions of neurons and non-neuronal cells in the brain tissues using a bioinformatics approach. Meanwhile, Braun et al. [4] fractionated neurons and non-neuronal cells using FACS, although the differences between the neuronal and non-neuronal fractions of the brain appeared to be negligible in comparison to the differences between tissues. Finally, the fourth database was recently published with the freely available analysis results (http://www.liga.uni-luebeck.de/buccal_brain_correlation_results/). Sommerer et al. [16] examined the correlations of 120 individuals whose prefrontal cortex tissue was obtained from a brain bank and their buccal samples. Another limitation of these previous databases is that genetic variants, particularly methylation quantitative trait loci (mQTL), exert a strong influence on DNA methylation [17, 18]. This implies that DNA methylation levels regulated by mQTLs may vary greatly among ancestral groups, as genetic variants depend on ancestry. Research institutes in the United Kingdom, Canada, the United States, and Germany developed the four databases that are currently available. Although the information regarding ancestry in the first two and the fourth databases was unclear, the majority of the third dataset’s samples are of Caucasian descent. If a researcher discovers an association between a phenotype and DNA methylation in peripheral samples from the Japanese population, for example, and wishes to examine the correlation with the DNA methylation level in brain tissues, the reliability of correlation results for the interpretation of data from a different ancestral group (in this case, Caucasian) would be questionable. However, it will be challenging to eliminate the entire mQTLs. To address this gap in knowledge, we have developed the fifth database based on Japanese subjects with living human brain samples using the same concept as that of the previous studies, and for the first time as the database for the Asian ancestry, namely, Asia Methylation Array apprizing for Experimental Comparison of Peripheral tissue & Gray matter (AMAZE-CpG) (https://snishit-amaze-cpg.web.app/). In the present study, after a preliminary evaluation of the results obtained from our dataset, we compared the effects of adjusting for cell-type proportion and the differences between IMAGE-CpG and the other first two databases derived from different ancestry.

## Materials and Methods

### Ethics statement

The study protocol was approved by the Ethics Committee of the University of Fukui, Japan (Assurance no. 20200028), Yamaguchi University School of Medicine, Japan (Assurance no. 2020–202), and Sugita Genpaku Memorial Obama Municipal Hospital (Assurance no. 2–7). Moreover, this study was carried out in accordance with the Declaration of Helsinki and the Ethical Guidelines for Clinical Studies of the Ministry of Health, Labour and Welfare of Japan. All participants provided either written informed consent or both informed consent and assent.

### Participants and sample collection

**Fig. 1:**
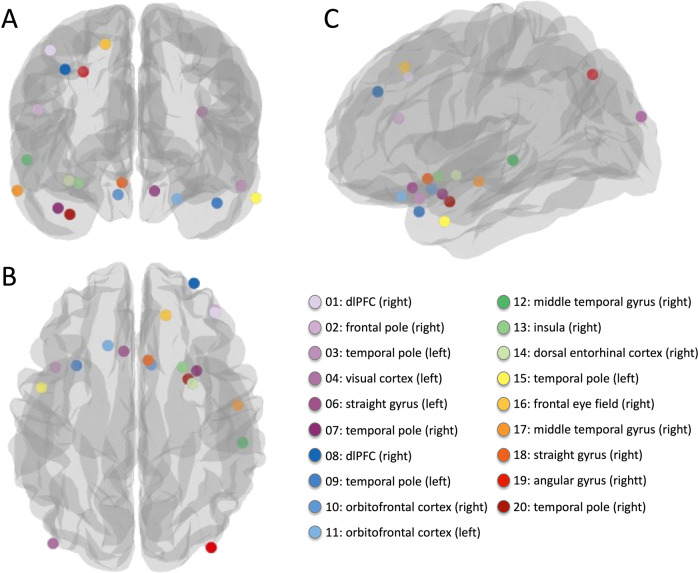
Location of each resected brain tissue according to the recorded Montreal Neurological Institute (MNI) coordinates. (**A**) front, (**B**) top, and (**C**) left-side views. Each sample is represented by a colored circle.

Twenty subjects undergoing neurosurgery for their clinical purposes, including intractable epilepsy, meningioma, and cerebrovascular diseases, were recruited for this study at the University of Fukui Hospital, Yamaguchi University Hospital, and Sugita Genpaku Memorial Obama Municipal Hospital (Table 1). Subjects were excluded if they had other serious concurrent genetic or physical diseases, but a patient no. 4 was included since the researchers noticed it later and the brain tissue was classified as normal tissue (Supplementary Table S1). A portion of each resected brain tissue was immediately cut into several pieces less than 5 mm^3^ and preserved in RNAlater® (Thermo Fisher Scientific, Inc., MA, US) for RNA and DNA stability and long-term storage. All Montreal Neurological Institute (MNI) coordinates corresponding to the regions of brain tissue resected were recorded by the primary surgeons for each case by clicking on the standard online brain image (https://neurosynth.org/locations/) (Table 1 and Fig. 1 created by the Python library, Nilearn [19]). We also used the novel online classification tool to confirm DNA methylation-based classification of central nervous system tumors (https://www.molecularneuropathology.org/mnp/) [7] (Supplementary Table S1) because the brain tissues used in the present study were not on a single disease, but across multiple diseases, and we intended to make explicit the potential impact of it. This tool classified 15 brain tissues as “Control tissues,” which means normal tissues. During the surgery, whole blood samples were collected in EDTA tubes and immediately preserved in RNAlater® (whole blood: RNAlater® = 5: 13). They were immediately stored overnight at 4 °C, then at −20 °C for long-term storage. Saliva samples were collected using the Oragene DISCOVER^TM^ kit (DNA Genotek Inc., Ottawa, CA, OGR-500) and stored at room temperature (RT) until DNA extraction at a later time. Buccal epithelial tissues (buccal) were collected using individually packaged commercial cotton swabs (four swabs/subject) and used for DNA extraction after air-drying at RT for a few days. The saliva and buccal swabs were collected 11.0 and 12.2 days after the operation, on average, respectively. For each sample, the date of acquisition in relation to the date of surgery was recorded.

**Table 1:** Subject and brain sample characteristics.

	Clinical demographics						Brain tissue		MNI coordinate			Day(s) after surgery	
ID	Sex	Age (y)	Height (cm)	Weight (kg)	BMI	Diagnosis	Brodmann areas	Hemisphere	X	Y	Z	Saliva	Buccal
**1**	male	69	160	37.5	14.6	primary central nervous system vasculitis	dlPFC	right	38	34	44	7	7
**2**	female	59	150	45.5	20.2	falx meningioma	frontal pole	right	42	41	18	3	3
**3**	male	60	164.5	66.8	24.7	internal carotid artery-posterior communicating artery aneurysm	temporal pole	left	−50	14	−18	11	11
**4**	female	73	157	40	16.2	metastatic brain tumor^a^ (breast cancer)	visual cortex	left	−45	−85	18	7	7
**6**	male	61	164	69.3	25.8	anterior communicating artery aneurysm	straight gyrus	left	−8	24	−19	16	8
**7**	male	69	169	57	20.0	internal carotid artery stenosis	temporal pole	right	38	13	−29	9	9
**8**	male	67	168	65	23.0	frontal lobe tumor	dlPFC	right	29	51	34	15	15
**9**	female	45	175.5	83	26.9	internal carotid artery stenosis	temporal pole	left	−38	16	−26	7	7
**10**	female	70	155	70	29.1	anterior communicating artery aneurysm	orbitofrontal cortex	right	9	16	−22	6	6
**11**	female	73	152	44.1	19.1	internal carotid artery-posterior communicating artery aneurysm	orbitofrontal cortex	left	−18	28	−22	15	9
**12**	male	60	172	63.9	21.6	tentorial meningioma	middle temporal gyrus	right	63	−32	−10	27	27
**13**	male	50	170	61	21.1	internal carotid artery-posterior communicating artery aneurysm	insula	right	28	14	−17	15	15
**14**	female	60	160.5	49.7	19.3	middle cerebral artery aneurysm	dorsal entorhinal cortex	right	34	4	−17	12	12
**15**	female	13	160	50	19.5	temporal lobe epilepsy, glioma	temporal pole	left	−60	2	−26	11	42
**16**	male	62	185	93.4	27.3	frontal lobe meningioma, epilepsy	frontal eye field	right	14	32	47	13	13
**17**	male	28	171	60	20.5	temporal lobe epilepsy	middle temporal gyrus	right	63	−9	−24	14	14
**18**	male	67	170	53.3	18.4	internal carotid artery stenosis	straight gyrus	right	7	18	−16	10	10
**19**	female	51	162	55	21.0	Subarachnoid hemorrhage, moyamoya disease	angular gyrus	right	36	−80	45	11	11
**20**	female	73	152.5	45.5	19.6	internal carotid‐posterior communicating aneurysm	temporal pole	right	33	8	−33	0	6

^a^Later noticed that the brain tumor was metastatic, but we included this subject according to the result of DNA methylation-based classification shown in supplementary Table 2.

Subject 5 was removed from downstream analyses due to mixed result seen in MDS plot.

### DNA extraction

Brain DNA was extracted using AllPrep DNA/RNA/miRNA Universal Kit (QIAGEN, Hilden, Germany) after disrupting and homogenizing 10 mg RNAlater® preserved brain tissues by a rotor–stator (speed: 6.5 m/sec, running time: 45 s, FastPrep FP120J-100, Savant Instruments, Inc.) with tissue homogenization beads (Lysing Matrix D, 2 mL, MP biomedicals, LLC, CA, US). Although this preprocessing was originally for RNA extraction, we followed the instructions in the RiboPure^TM^ RNA Purification Kit (Thermo Fisher Scientific, Inc., MA, US) for preprocessing for DNA extraction using RNAlater® preserved blood samples. In brief, the RNAlater® preserved blood samples (720 μL) were centrifuged for 1 min at 16 000 ×*g*, and the supernatant was removed. Then, 200 μL of PBS was pipetted in and mixed, and centrifugation and supernatant removal were repeated. Finally, 200 μL of PBS was added and pipetted together before being used for DNA extraction. QIAamp DNA Mini kit (QIAGEN, Hilden, Germany) was used to extract DNA from blood and buccal, and the protocols for each tissue type were followed. Meanwhile, the prepIT®•L2P reagent (DNA Genotek Inc., Ottawa, CA) was used to extract DNA from saliva. The DNA yield was determined using the Qubit^TM^ dsDNA High Sensitivity Assay Kit (Thermo Fisher Scientific, Inc., MA, US) [8].

### DNA methylation array and pre-processing

For each sample, 500 ng of DNA was bisulfite converted with the EZ DNA Methylation^TM^ Kit (Zymo Research, D5002). Meanwhile, the Infinium HumanMethylationEPIC BeadChip Kit (Illumina, WG-317-1002) array was used to assess genome-wide DNA methylation. Samples were grouped by individuals and randomized onto the chips. The arrays were scanned with the Illumina iScan platform.

To allow for fair comparison with the previous study [4], the DNA methylation dataset was pre-processed using the R packages Minfi [20, 21] and RnBeads [22]. Background correction was performed with the Noob method in Minfi. Probes were filtered out using RnBeads if they: (1) overlapped within 5 bp of an SNP (21 361 probes); (2) had a detection *P*-value > 0.01 or were deemed unreliable measures based on RnBeads’s greedy-cut algorithm (16 367 probes); or (3) were context-specific sites (probes other than those for CpG methylation; 2 873 probes). Probes excluded with overlapping SNPs were assigned by RnBeads using the version of dbSNP derived from Genome Reference Consortium Human Build 37 patch release 10 (GRCh37.p10). These SNPs could disrupt the probe binding at the target sites and artificially lower the intensity signals that may make results inconsistent [23]. With the application of these filters, 825 637 probes were included in the dataset. Beta mixture quantile dilation (BMIQ) was used to normalize the samples.

### IMAGE-CpG dataset (GSE111165) pre-processing

The GSE111165 dataset was similarly pre-processed. This dataset has both 450 K and EPIC data, but we used only the EPIC data. Minfi’s Noob method was used to correct the background. Using RnBeads, we filtered out probes if they: (1) overlapped within 5 bp of an SNP (21 358 probes); (2) had a detection *P*-value > 0.01 or were deemed unreliable measures based on RnBeads’s greedy-cut algorithm (10 053 probes); or (3) were context-specific sites (2 894 probes). After filtering, we obtained 831 786 probes for the dataset. BMIQ was used to normalize the samples.

### Pre-processing for GSE59685 [12] and GSE95049 [13] datasets

The pre-processing was similar to those of the original studies [12, 13]. The total number of probes and samples for GSE59685 and GSE95049 was 437 649 and 67 for brain tissues (PFC, EC, STG, and CER) and blood, and 444 283 and 15 samples of brain tissues (BA10, BA20, and BA7) and blood, respectively. The R code is available as Supplementary Material.

### Estimation of ancestral data

To confirm the ancestral differences between the datasets, we generated ancestry principal components (PCs) from blood DNA methylation using the method of Barfield et al. [24].

### Cellular composition adjustment

Given that cellular heterogeneity affects methylation, the AMAZE-CpG and IMAGE-CpG datasets were pre-processed with the adjustment (Adj) in parallel, as Edgar et al. [13] did, in addition to the raw dataset (Raw). Cellular heterogeneity was predicted using CETS [25] and EpiDISH [15] for the brain and other peripheral tissues, respectively (Supplementary Table S2). Brain tissue methylation was adjusted by the proportion of neurons. Only five cell type values (B, NK, CD4T, CD8T, and Mono, without Neutro) were used to adjust blood methylation [26], because they lie in the [0,1] range and are constrained to sum to 1 within a sample; including all six values as covariates would induce multicollinearity [27]. Saliva and buccal tissue methylations were adjusted by the proportion of epithelial cells. We processed both Raw and Adj datasets in each analysis to compare their performance.

### Statistical analysis

All statistical analyses were performed in R [28]. Two approaches to cross-tissue correlation were used. First, Pearson’s correlation was used to calculate overall levels of DNA methylation correlation from the average methylation across subjects for each tissue. For the overall correlation, all 825 637 (AMAZE-CpG) and 831 786 (IMAGE-CpG) CpGs were used in the calculation. Second, a within-subject method was employed. Because of the small sample size and the possible inappropriate influence of outliners on the correlation coefficient, a correlation coefficient (*rho*) and its significance level were calculated for each individual CpG using a non-parametric Spearman’s rank correlation test. Variable CpGs were classified as Hannon et al. [12] previously defined. This method involved excluding DNA methylation values in the upper and lower 10th percentile for each CpG, then classifying as variables those CpGs with a remaining range difference of at least 5%. Because the number of these variable CpGs varies from tissue to tissue, the correlation analyses between tissues were limited to the CpGs found to be variable in all four tissues (AMAZE-CpG: 287 033 CpGs _[Raw]_ and 189 704 CpGs _[Adj]_, and IMAGE-CpG: 280 302 CpGs _[Raw]_ and 194 310 CpGs _[Adj]_). Furthermore, cross-database correlation analyses were conducted to demonstrate the potential similarities and differences in the correlation coefficients of each dataset. In this case, 815 541 for the entire dataset and 233 904 _[Raw]_ and 136 929 _[Adj]_ for CpGs found to be variable in both datasets were included in the analysis. CpG sites with an absolute difference in *rho* between AMAZE-CpG and IMAGE-CpG lesser (greater) than 0.2 were defined as less (more) dependent on the differences between the datasets which include the differences of ancestry, age, brain regions, or cellular composition.

### Assessment of potential SNP confounding effect

As described in Supplementary Methods, we developed filtering parameters based on our dataset to identify probes that may be affected by SNPs.

### mQTL classification

A list of mQTL (http://www.mqtldb.org/) with Gaunt et al.’s original *P*-value cutoff of *P* < 1 × 10^−14^ [29], yielding 27 623 and 27 748 CpGs under genetic influence, which overlapped with the AMAZE-CpG and IMAGE-CpG datasets, respectively.

## Results

**Fig. 2:**
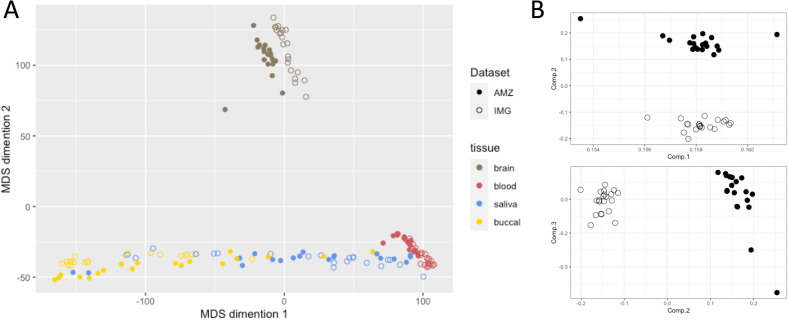
Tissue specificity and ancestral differences in genome-wide DNA methylation between the AMAZE-CpG and IMAGE-CpG datasets. **A** Multidimensional scaling (MDS) of genome-wide DNA methylation levels from brain, blood, saliva, and buccal samples using a Euclidian distance plot. **B** Principal components (PCs) for population stratification based on DNA methylation. Top: First and second PC from PC_0bp_. Bottom: Second and third PC from PC_0bp_. Solid circles represent the AMAZE-CpG dataset, whereas open circles represent the IMAGE-CpG dataset.

The degree of similarity in genome-wide DNA methylation between the brain and peripheral tissues was determined using a multidimensional scaling (MDS) plot (Fig. 2A). Our data’s MDS plot revealed a similar pattern to that of the previous study [4]. The MDS plot revealed the brain samples clustering separately from all peripheral tissues, whereas the peripheral tissues clustering is as predicted by their cellular compositions. One sample of brain tissue was plotted at intermediate region between the brain and blood clusters, and contamination was suspected. Upon visual inspection, the specific brain tissue appeared as if both the brain and blood had been burned with an electrocautery scalpel. Consequently, the subject was eliminated from further analysis. An analysis of DNA methylation-based principal components for population stratification revealed objectively that our dataset’s population was ancestrally distinct from the IMAGE-CpG dataset’s population (Fig. 2B).

As previously reported [4], we evaluated cross-tissue DNA methylation correlations in two different ways. The first method averaged DNA methylation across subjects, whereas the second focused on a within-subject analysis of individual CpGs. The former is a global representation of all CpGs present on the array. For each tissue, the methylation values were averaged across all subjects, following which, the correlation of all CpGs between two tissues was calculated. The latter is an individual CpG-centric strategy. The correlation was calculated separately for each CpG using distinct data points from each subject.

### Across-subject correlations

**Fig. 3:**
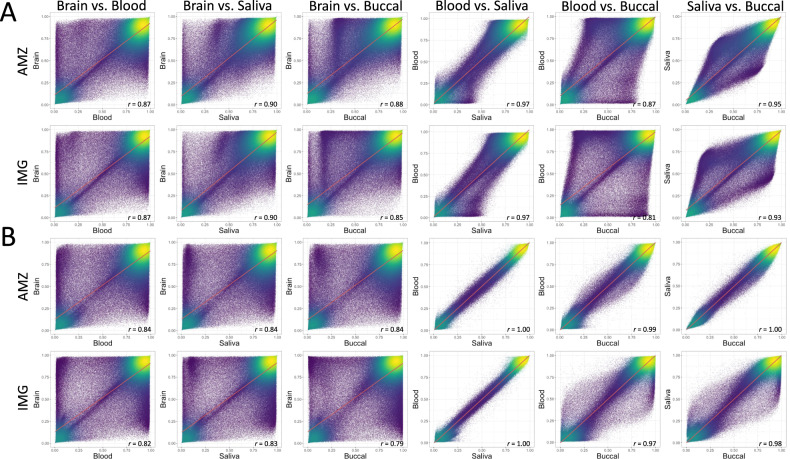
Density scatter plots of cross-tissue correlation for each tissue combination. (**A**) Raw, and (**B**) cell proportion adjusted datasets. AMZ: AMAZE-CpG dataset, and IMG: IMAGE-CpG dataset. Red line: regression line.

Overall levels of genome-wide DNA methylation correlation were calculated from the average methylation across all CpGs between each peripheral tissue and brain using Pearson’s correlation (Full: Fig. 3, and Variable CpGs: Supplementary Figure S1). Blood and saliva showed the highest correlation for DNA methylation (*r* = 0.97 _[Raw]_ and *r* = 1.00 _[Adj]_), followed by saliva and buccal (*r* = 0.95 _[Raw]_ and *r* = 1.00 _[Adj]_), and finally blood and buccal (*r* = 0.87 _[Raw]_ and *r* = 0.99 _[Adj]_). When peripheral tissues were compared to brain, relatively high levels of correlation were observed (blood–brain: *r* = 0.87 _[Raw]_ and *r* = 0.84 _[Adj]_; saliva–brain: *r* = 0.90 _[Raw]_ and *r* = 0.84 _[Adj]_; and buccal–brain *r* = 0.88 _[Raw]_ and *r* = 0.84 _[Adj]_).

### Within-subject correlations at each CpG

**Fig. 4:**
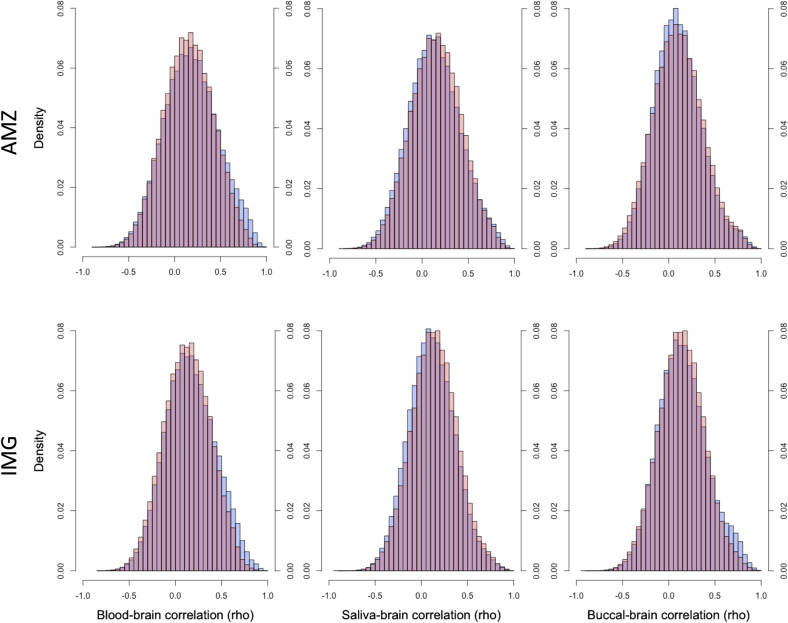
Histograms depicting the distribution of correlations (*rho*) of the individual CpGs between the brain and blood (left), saliva (center), and buccal (right). (red) Datasets in raw and (blue) cell proportion adjusted form. AMZ AMAZE-CpG dataset, and IMG IMAGE-CpG dataset.

A within-subject comparison at the individual CpG level revealed only a fraction of CpGs having significant correlations between the peripheral tissues and the brain. Of the 825 637 CpGs, 19.0% (156 999 CpGs) _[Raw]_ and 14.7% (121 321 CpGs) _[Adj]_ were correlated at a nominal level of significance (*P* < 0.05, uncorrected) in blood; 14.4% (119 294 CpGs) _[Raw]_ and 14.5% (119 748 CpGs) _[Adj]_ in saliva; and 9.8% (80 580 CpGs) _[Raw]_ and 11.1% (91 625 CpGs) _[Adj]_ in buccal, each with an *r* >± 0.46. Meanwhile, moderately strong correlations (*r* > 0.5) were seen in 14.7% _[Raw]_ and 10.6% _[Adj]_ of the CpGs in blood, 10.5% _[Raw]_ and 10.5% _[Adj]_ in saliva, and 6.8% _[Raw]_ and 7.4% _[Adj]_ in buccal. The mean and median correlations for blood were *r*_mean_ = 0.18 _[Raw]_ and *r*_mean_ = 0.16 _[Adj]_, and *r*_median_ = 0.17 _[Raw]_ and *r*_median_ = 0.15 _[Adj]_, respectively; for saliva, *r*_mean_ = 0.14 _[Raw]_ and *r*_mean_ = 0.15 _[Adj]_, and *r*_median_ = 0.13 _[Raw]_ and *r*_median_ = 0.15 _[Adj]_; and for buccal, *r*_mean_ = 0.10 _[Raw]_ and *r*_mean_ = 0.10 _[Adj]_, and *r*_median_ = 0.09 _[Raw]_ and *r*_median_ = 0.10 _[Adj]_. Figure 4 shows the distribution of correlations for each peripheral tissue’s correlation to the brain across all CpGs. The variable CpGs for each peripheral tissue is as follows: blood = 365 648 _[Raw]_ and 237 715 _[Adj]_; saliva = 456 453 _[Raw]_ and 335 478 _[Adj]_; and buccal = 453 651 _[Raw]_ and 349 680 _[Adj]_. Of the variable CpGs, with variability calculated as described by Hannon et al. [12], 19.6% _[Raw]_ and 20.0% _[Adj]_ of the variable CpGs in blood, 15.5% _[Raw]_ and 21.9% _[Adj]_ in saliva, and 13.1% _[Raw]_ and 29.4% _[Adj]_ in buccal were nominally correlated. Additionally, 15.4% _[Raw]_ and 16.0% _[Adj]_ of the variable CpGs in blood, 12.1% _[Raw]_ and 17.6% _[Adj]_ in saliva, and 10.5% _[Raw]_ and 15.8% _[Adj]_ in buccal were moderately correlated (*r* > 0.5). These within-subject comparisons contrast with the correlations seen across the average methylation values for all CpGs, where blood had the greatest correlation. A portion of the individual CpGs survived the Benjamini–Hochberg correction for multiple testing. This included 53 482 CpGs _[Raw]_ and 13 064 CpGs _[Adj]_ for blood, 26 576 CpGs _[Raw]_ and 19 584 CpGs _[Adj]_ for saliva, and 14 764 CpGs _[Raw]_ and 14 214 CpGs _[Adj]_ for buccal, with corresponding *rho* values greater than 0.65 (Supplementary Table S4). We choose the Benjamini–Hochberg correction instead of the Bonferroni correction since only the *P* = 0 probe (The *P*-values of the Spearman correlation analysis were calculated as 0 if less than 7.57e-08. See Supplementary Table S4, column F.) were listed if the Bonferroni correction was used.

### Correlations by subject

We evaluated the consistency of the correlations among individuals. For all but seven of the 19 subjects, the saliva–brain correlation (*r*_*mean*_ = 0.86 _[Raw]_) was greater than the blood–brain correlation (*r*_*mean*_ = 0.85 _[Raw]_). The buccal–brain correlation (*r*_*mean*_ = 0.84 _[Raw]_) was the lowest in every individual, except for nine individuals for whom it was higher than blood–brain and seven individuals for whom it was higher than saliva–brain (Supplementary Fig. S2). As Braun et al. [4] discovered, our data also replicated such phenomenon that when the buccal–brain correlation increased within subjects, their buccal–blood correlations also increased (*r* = 0.81, *P* = 2.9e − 05 _[Raw]_) (Supplementary Fig. S3). This was not the case for the remaining correlational combinations.

### Cross-database correlations of the correlation coefficients with IMAGE-CpG

**Fig. 5:**
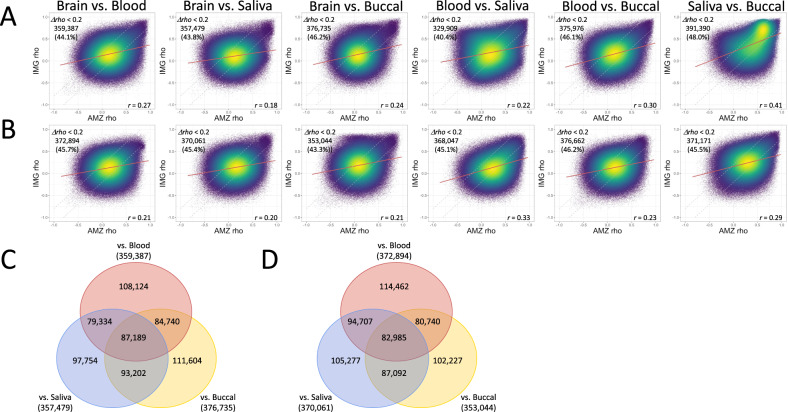
Comparison of correlation coefficients between the datasets. Correlation density scatter plots of the Spearman’s *rho* between the datasets for each tissue combination for (**A**) raw and (**B**) cell proportion adjusted datasets. Venn diagrams summarizing the number of correlations between the brain and each peripheral tissue that did not differ within *Δrho* < 0.2 between the datasets for (**C**) Raw and (**D**) cell proportion adjusted datasets. AMZ AMAZE-CpG dataset, and IMG IMAGE-CpG dataset. The regression line is in red. The dashed line represents the boundary line within *Δrho* < 0.2 between the datasets.

Comparing peripheral tissues across datasets, saliva and buccal had the highest correlation coefficients (*r* = 0.41 _[Raw]_ and *r* = 0.29 _[Adj]_), followed by blood and buccal (*r* = 0.30 _[Raw]_ and *r* = 0.23 _[Adj]_), and finally blood and saliva (*r* = 0.22 _[Raw]_ and *r* = 0.33 _[Adj]_) (Full: Fig. 5, and Variable CpGs: Supplementary Fig. S4). When comparing peripheral tissues to the brain, we determine relatively lower levels of correlation between the datasets (blood–brain *r* = 0.27 _[Raw]_ and *r* = 0.21 _[Adj]_, buccal–brain *r* = 0.24 _[Raw]_ and *r* = 0.21 _[Adj]_, and saliva–brain *r* = 0.18 _[Raw]_ and *r* = 0.20 _[Adj]_). We tentatively defined the methylation sites for which the difference in correlation coefficients was less than 0.2, as relatively common probes regardless of database differences. This means that they are stable and relatively less dependent on the differences between datasets. The number of sites varied by tissue type, with 87 189 _[Raw]_ and 82 985 _[Adj]_ sites being shared by all three types.

### Correlation coefficients across databases for the GSE59685 [12] and GSE95049 [13] datasets

Both GSE59685 and GSE95049 datasets exhibited correlations across subjects (Supplementary Fig. S5). In each case, the correlation coefficients between AMAZE-CpG and the two previous databases were relatively lower (Supplementary Fig. S6).

### Evaluation of the potential SNP confounding effect

The results obtained from gap statistics and K-means method parameters are summarized in Supplementary Table S3. Based on the criteria, SNPs were likely to affect 6 662 in blood, 27 487 in saliva, 19 280 in buccal, and 4 780 in all three common samples. Supplementary Fig. S7 shows that the cluster number in each case was 1, 2, and 3. The numbers 2 and 3 are examples of CpG sites that were affected by SNPs.

### Correlation of psychiatric-associated genes

As reported by Braun et al. [4], the brain–peripheral correlations of candidate psychiatric genes were investigated (Supplementary Fig. S8). Variable levels of correlation among the peripheral tissues were revealed for each gene, with across-subject correlations ranging *rho* = 0.79–0.99 and within-subject correlations ranging *rho* = 0.01–0.36 for the individual CpGs, with the highest peripheral correlations varying by gene (Supplementary Table S5).

### AMAZE-CpG

A website for AMAZE-CpG has been created that enables researchers to examine DNA methylation levels and the degree of correlation between individual CpGs and genes from the Illumina EPIC platform (19 Japanese subjects; brain, blood, saliva, and buccal). It can be accessed at https://snishit-amaze-cpg.web.app/.

## Discussion

The results of cross-tissue correlations in AMAZE-CpG and IMAGE-CpG were quite similar. However, cross-database correlations of the correlation coefficients with IMAGE-CpG when comparing peripheral tissues to the brain were relatively low (*r* = 0.18–0.27 _[Raw]_). This could be attributed to ancestral differences, but it was also likely influenced by population differences (e.g., age and individual differences) and differences in brain regions and disease types. It may be helpful to examine whether a correlation exists with the brain using either of the two databases for relatively common probes where the difference in correlation coefficients is less than 0.2, as we tentatively defined. When interpreting data from Japanese and Asians, AMAZE-CpG would be more appropriate for probes greater than 0.2. The output results of the AMAZE-CpG also include whether the difference in correlation coefficient with the IMAGE-CpG is less than 0.2. The correlation coefficients between AMAZE-CpG and the two preceding databases, Hannon et al. [12] and Edgar et al. [13], were lower than those between AMAZE-CpG and IMAGE-CpG. This could be due to the different microarray formats (450 K) and the use of post-mortem brains. When the case was compared to the IMAGE-CpG dataset, relatively similar trends were observed, indicating that those influences were more significant than differences in ancestry or other factors. Therefore, when interpreting the results of individual studies using these databases, deciding which to use will be crucial for more accurate interpretation.

We examined correlations restricted to variable CpGs using Hannon et al.’s [12] definitions and found that the correlation coefficients dropped significantly, similar to those in Braun et al. [4]. This is most likely due to the effect of non-variable CpGs or static methylation sites, many of which are expected to be dependent on genetic sequences, and are based on removing the correlation that appeared quasi-strong between the tissues. Furthermore, even when variables were limited to variable CpGs, the methylation correlation between brain and peripheral tissues was not significantly improved. According to the current data, the majority of methylation in the brain is most likely not synchronized with methylation in the periphery. Despite this, variable CpGs that correlate in the brain and periphery, although in small numbers, may have biological relevance and could be useful for inferring brain methylation from peripheral tissues.

Previously, Edgar et al. [13] constructed a dataset that was adjusted for cell proportion and examined the methylation correlation between brain and blood. We followed suit and examined both raw and adjusted datasets, but the correlation coefficients between brain and peripheral methylation did not differ dramatically regardless of cell proportion adjustment. This effect was especially noticeable for the correlation coefficients between blood and saliva methylation and between saliva and buccal methylation. Saliva DNA originates from a mixture of neutrophils and buccal epithelial cells [8]. Therefore, adjusting for these influences would most likely bring it closer to one of these properties. Individual methylation studies may benefit from this cell proportion adjustment to unify their disparate use of blood, saliva, or buccal. For example, when the study subjects are not clinical populations or children, many studies use saliva or buccal, rather than blood, as the source of DNA [8]. More studies will use the buccal if the subjects are even younger children, such as neonates or infants [30]. This means that the tissues analyzed for methylation differ from study to study, making comparison of study results difficult. Given that adjusting for cell proportion can result in such a high correlation between peripheral tissues, there may be a way to account for minor tissue differences in blood, saliva, and buccal.

In a recent study, we found that the promoter region of the *OXT* gene methylation in saliva DNA was more common in children who experienced maltreatment than in controls [31]. Given the tissue specificity issue in this study, we used IMAGE-CpG to demonstrate that correlation with the brain was not found. However, when the same locations were tested with AMAZE-CpG, these saliva methylations were positively correlated with brain methylation in multiple locations (Supplementary Table S6). This difference may or may not have been the result of using an ancestry-matched database, but it is an example of how an ancestry-matched database can be more reliable.

This study has six major limitations. First, the sample size is still smaller than three of the previously published four datasets, which may make the dataset underpowered for tissue correlations of DNA methylation patterns. We will further increase the sample size to eliminate the impact of individual differences. Because we have the MNI coordinates for all of the brain tissues used in this study, we may be able to perform correlation analysis focusing on each exact brain region if the sample size is increased. The CER had a significantly different methylation pattern than other brain regions in the dataset of Hannon et al. [12]; thus, it is preferable to analyze the data by brain region. However, because this was a post-mortem brain study, the rough brain regions (frontal and temporal cortexes, etc.) can be chosen from the listed biobanks. While the present study has the strength of being able to handle living samples, it has the weakness of not being able to choose the region. Second, the subjects of the database underwent neurosurgery for multiple purposes. We did not address the potential disease-driven differences, although most of the brain tissues were classified as “Control tissues (normal tissues)” by the online classification tool for DNA methylation-based classification of central nervous system tumors. This also overlaps with the limitations of the sample size, however, as more sample numbers are accumulated, the potential differences driven by each disease should also be clarified. Third, although brain tissues contain neurons and various types of glial cells, we did not fractionate them using FACS, as Braun et al. [4] did. After cell sorting, it would be useful to profile the methylation of each brain cell type. Forth, even though the influence of SNPs was eliminated using methods based on gap statistics and k-means clustering, not everything was accurately captured. Our correlation analysis should be less susceptible than parametric correlation analysis, even if a few samples are outliners, because of the nonparametric Spearman’s analysis. The parameters we used to filter the potential CpG sites influenced by the SNPs are included in the database search results output. Therefore, even if the correlation coefficient appears to be high, it should be interpreted appropriately by referring to these parameters to determine whether it is due to SNP influences or not. Fifth, population stratification was needed to precisely determine by genotyped data to clarify the ancestral background, although at least we have estimated it based on DNA methylation. Indirectly, it also links to the analysis of mQTLs. It will be an important approach for future investigations. Finally, the methylation targeted by this study accounts for only about 3% of whole genome methylation (28 million). Future studies must transition from microarray-based analysis to whole genome sequencing.

In conclusion, this study has developed the first Asian population genome-wide DNA methylation dataset as a brain–peripheral tissues correlation database. This database would be especially useful in interpreting individual epigenetic study results from various Asian populations, including those from Japan. Although peripheral methylation status does not always reflect that of the brain, it cannot be applied universally, and some may reflect brain methylation. If we can narrow in on such methylation, neuroepigenetics will be accelerated, and the current database will contribute to them.

## Supplementary information

Supplementary information

Supplementary Table S2

Supplementary Table S4

Supplementary Table S5

Supplementary Methods

Code1

Code2

Dataset 1

## Data Availability

The DNA methylation microarray data that support the study’s findings have been deposited in the Gene Expression Omnibus database (GEO) under the primary accession code GSE214901.

## Code availability

The R code for GSE59685 [12] and GSE95049 [13] datasets pre-processing is available as Supplementary Material.
