# Supplementary material for: Cross-tissue correlations of genome-wide DNA methylation in Japanese live human brain and blood, saliva, and buccal epithelial tissues
Source: Transl Psychiatry. 2023 Feb 27;13:72. doi: 10.1038/s41398-023-02370-0 (PMC9968710; doi:10.1038/s41398-023-02370-0)
Supplement: Supplementary file 1 — Supplementary information [file 41398_2023_2370_MOESM1_ESM.docx]

**Supplementary Table S1.** DNA methylation-based classification of central nervous system tumors.



| **ID** | **Classification** | **Score** |
| --- | --- | --- |
| **1** | Control tissues | 0.89 |
| **2** | Control tissues | 1.00 |
| **3** | Low-grade glial/glioneuronal/neuroepithelial tumors | 0.43 |
| **4** | Control tissues | 1.00 |
| **6** | Control tissues | 0.93 |
| **7** | Control tissues | 0.98 |
| **8** | Ependymal tumors | 0.38 |
| **9** | Control tissues | 0.66 |
| **10** | Control tissues | 0.83 |
| **11** | Control tissues | 1.00 |
| **12** | Control tissues | 1.00 |
| **13** | NA | 0.10 |
| **14** | Control tissues | 0.98 |
| **15** | Pediatric-type diffuse high-grade gliomas | 0.49 |
| **16** | Control tissues | 0.85 |
| **17** | Control tissues | 0.91 |
| **18** | Control tissues | 0.98 |
| **19** | Control tissues | 1.00 |
| **20** | Control tissues | 0.42 |

**Supplementary Figure S1.** Cross-tissue correlation density scatter plots for each tissue combination in variable CpGs. (A) Raw, and (B) cell proportion adjusted datasets. AMZ: AMAZE-CpG dataset, and IMG: IMAGE-CpG dataset. Red line: regression line.

**Supplementary Figure S2.** Degree of DNA methylation correlation of all CpG probes between each peripheral tissue and brain for all subjects.

**Supplementary Figure S3.** (A) Calculated distance from the MDS plots between each peripheral tissue and its corresponding brain sample in AMAZE-CpG dataset is plotted against the level of correlation among those samples. (B) Relationship between the MDS distance of peripheral-brain and peripheral–another peripheral.

**Supplementary Figure S4.** Comparison of correlation coefficients in variable CpGs between the datasets**.** Correlation density scatter plots of spearman’s *rho* between the datasets for each tissue combination for (A) Raw, and (B) cell proportion adjusted datasets. AMZ: AMAZE-CpG dataset, and IMG: IMAGE-CpG dataset. Red line: regression line. Dashed line: the border line within *Δrho* < 0.2 between the datasets.

**Supplementary Figure S5.** Density scatter plots between each tissue from the tissues’ average methylation from GSE59685 (Left) and GSE95049 (Right). (A) Raw beta and (B) adjusted beta by cell proportion.

**Supplementary Figure S6.** Density scatter plots between AMZ or IMG *rho* and GSE59685 (Left) and GSE95049 (Right) between each tissue from the individual methylation. (A) Raw beta and (B) adjusted beta by cell proportion.

**Supplementary Table S3.** Proportion of probes evaluated as potentially affected by SNPs



| **Brain vs** | **Blood** | | **Saliva** | | **Buccal** | | **Common** | |
| --- | --- | --- | --- | --- | --- | --- | --- | --- |
| **Est. cluster size** | **# of probes (%)** | **Between>1** | **# of probes (%)** | **Between>1** | **# of probes (%)** | **Between>1** | **# of probes (%)** | **Between>1** |
| 1  2  3 | 566289 (68.6)  129185 (15.6)  130163 (15.8) | 115  3813  2734 | 541321 (65.6)  114593 (13.9))  169723 (20.5) | 5521  4586  17380 | 544061 (65.9)  135835 (16.4)  145741 (17.7) | 3597  6925  8758 | 383890  41265  39414 | 33  3184  1563 |
|  | 825637 (100.0) | 6662 | 825637 (100.0) | 27487 | 825637 (100.0) | 19280 | 464569 (56.3) | 4780 |

**Supplementary Figure S7.** Examples for each estimated cluster cases (A: 1 cluster, B:2 clusters, and C:3 clusters). Top: scatter plots between blood and brain methylation, middle: gap-k statistics plots, bottom: clustering plots using K-means method

**Supplementary Figure S8.** Average DNA methylation (β) patterns in AMAZE-CpG dataset in candidate psychiatric genes across brain, blood, saliva, and buccal. (A) FK506 binding protein 5 (*FKBP5*), (B) the glucocorticoid receptor (*NR3C1*), (C) brain-derived neurotrophic factor (*BDNF*), (D) the serotonin transporter (*SLC6A4*), (E) aryl-hydrocarbon receptor repressor (*AHRR*), (F) spindle and kinetochore associated complex subunit 2 (*SKA2*), (G) corticotropin-releasing hormone (*CHR*), and (H) oxytocin (*OXT*).

**Supplementary Table S6.** *OXT* methylation correlations between brain and saliva from the AMAZE-CpG database

|  |  | **Raw** | | **Adj** | |  |  |  |  |  |  |
| --- | --- | --- | --- | --- | --- | --- | --- | --- | --- | --- | --- |
| **Probe ID** | **Chr:MAPINFO** | ***rho*** | ***P*** | ***rho*** | ***P*** | **SNP_SA** | **mQTL_flag** | **varSA_flag** | **varSAadj_flag** | **SA_A-I0.2_flag** | **SAadj_A-I0.2_flag** |
| cg09129163 | Chr20:3050786 | 0.63 | 0.005 | 0.29 | 0.22 | 0 | FALSE | TRUE | FALSE | FALSE | FALSE |
| cg07597882 | Chr20:3051493 | 0.37 | 0.12 | 0.13 | 0.60 | 0 | FALSE | TRUE | TRUE | FALSE | FALSE |
| cg04528380 | Chr20:3051528 | 0.32 | 0.18 | 0.08 | 0.73 | 0 | FALSE | TRUE | TRUE | FALSE | FALSE |
| cg04731988 | Chr20:3051954 | 0.28 | 0.24 | 0.29 | 0.23 | 0 | FALSE | TRUE | TRUE | FALSE | FALSE |
| **cg19776589** | Chr20:3052058 | 0.26 | 0.27 | 0.23 | 0.35 | 0 | FALSE | TRUE | TRUE | FALSE | TRUE |
| **cg07747220** | Chr20:3052115 | 0.53 | 0.02 | 0.19 | 0.42 | 0 | FALSE | TRUE | TRUE | FALSE | TRUE |
| **cg16887334** | Chr20:3052151 | 0.31 | 0.20 | 0.43 | 0.07 | 0 | FALSE | TRUE | TRUE | FALSE | TRUE |
| **cg13285174** | Chr20:3052221 | 0.32 | 0.18 | 0.25 | 0.29 | 0 | FALSE | TRUE | TRUE | FALSE | FALSE |
| **cg26267561** | Chr20:3052224 | 0.16 | 0.50 | 0.06 | 0.80 | 0 | FALSE | TRUE | TRUE | FALSE | FALSE |
| **cg01644611** | Chr20:3052253 | 0.37 | 0.12 | 0.27 | 0.26 | 0 | FALSE | TRUE | TRUE | FALSE | FALSE |
| **cg13725599** | Chr20:3052262 | 0.23 | 0.34 | 0.33 | 0.17 | 0 | FALSE | TRUE | TRUE | FALSE | FALSE |
| cg19592472* | Chr20:3052274 | 0.39 | 0.10 | 0.34 | 0.16 | 0 | FALSE | TRUE | TRUE | FALSE | TRUE |
| **cg26955850** | Chr20:3052345 | 0.28 | 0.24 | 0.44 | 0.06 | 0 | FALSE | TRUE | TRUE | TRUE | TRUE |
| cg09774842 | Chr20:3052483 | 0.38 | 0.11 | 0.47 | 0.05 | 0 | FALSE | FALSE | FALSE | FALSE | FALSE |
| cg06404175 | Chr20:3052692 | 0.83 | 6.4e-06 | 0.84 | 0 | 0 | FALSE | FALSE | FALSE | FALSE | FALSE |
| cg12099952 | Chr20:3053037 | 0.03 | 0.90 | -0.25 | 0.30 | 0 | FALSE | FALSE | FALSE | TRUE | FALSE |

**Bold**: probes constructed *OXT*mi (Nishitani S, et al., 2021)

*: removed by quality control process
