# Supplementary material for: Cross-tissue correlations of genome-wide DNA methylation in Japanese live human brain and blood, saliva, and buccal epithelial tissues
Source: Transl Psychiatry. 2023 Feb 27;13:72. doi: 10.1038/s41398-023-02370-0 (PMC9968710; doi:10.1038/s41398-023-02370-0)
Supplement: Supplementary file 5 — Supplementary Methods [file 41398_2023_2370_MOESM5_ESM.docx]

**Supplementary Methods**

Assessment of potential SNP confounding effect

Hannon et al. [10] raised the confounding effect issue of SNPs on DNA methylation correlation between the brain and peripheral tissues from the start. When an SNP strongly influences a probe, the positive correlation appears extremely strong, leading to misinterpretation. We removed probes that overlapped within 5 bp of an SNP during pre-processing, but this does not eliminate all SNP’s influence. Some pre-processing excludes or flags the probes that are already known to be affected by SNPs [4], but this does not cover them all. Furthermore, the effects may differ depending on the population. Therefore, instead of extrapolating from other data, we developed filtering parameters based on our dataset to identify probes that may be affected by SNPs. First, the number of clusters was determined mechanically using gap statistics. The number of clusters is determined to be 1, 2, or 3. In the case of 1, SNPs may have no influence. In the case of 2, two types of clusters are seen. Due to this database’s small sample size, it is assumed that not all three genotypes will appear, as some genotypes will likely appear infrequently. In the case of 3, this can result in the formation of clusters that may represent three different genotypes. However, even if it is determined to be 2 or 3, this would not be sufficient to ensure that the SNP caused the effect. Therefore, as another step, the distance between clusters was measured using the K-means method when the number of clusters was assumed to be 2 or 3. If the gap statistics were 2 or 3, and the distance between the clusters was greater than a certain value (>1), the SNPs would most likely be influencing. Even when the number of clusters is determined to be 1 based on the gap statistics, in some exceptional cases, the influence of SNPs is apparent by visual inspection. As such, even if the cases were determined to be cluster 1, those with a distance measured using the K-means method that exceeded 1, whether calculated under the assumption of clusters 2 or 3, were considered to be affected by SNPs.
